# Enthesitis in IBD Patients

**DOI:** 10.3390/jcm13154540

**Published:** 2024-08-03

**Authors:** Ivna Akrapovic Olic, Jonatan Vukovic, Mislav Radic, Zeljko Sundov

**Affiliations:** 1 Internal Medicine Department, University of Split School of Medicine, Soltanska 2, 21000 Split, Croatia; jonatan.vukovic@mefst.hr (J.V.); mislavradic@gmail.com (M.R.); zsundov@gmail.com (Z.S.); 2 Gastroenterology and Hepatology Department, University Hospital of Split, Spinciceva 1, 21000 Split, Croatia; 3 Internal Medicine Department, Rheumatology, Allergology and Clinical Immunology Division, University Hospital of Split, Spinciceva 1, 21000 Split, Croatia

**Keywords:** inflammatory bowel disease, Crohn’s disease, ulcerative colitis, extraintestinal manifestations, enthesitis

## Abstract

Inflammatory bowel disease (IBD) is marked by chronic inflammation of the gastrointestinal tract and encompasses two major subtypes, Crohn’s disease (CD) and ulcerative colitis (UC). IBD is frequently accompanied by extraintestinal manifestations (EIMs), with axial and peripheral spondyloarthritis (SpA) being the most common. Enthesitis, an inflammation of the bone insertions of capsules, ligaments, and tendons, represents an initial lesion in SpA. However, enthesitis remains an underestimated and often obscured EIM. The early detection of subclinical entheseal involvement in IBD patients using ultrasound (US) could provide an opportunity for timely intervention. US is a more feasible and affordable approach than magnetic resonance imaging (MRI). While previous meta-analyses have reported on the incidence and prevalence of SpA in IBD, specific attention to enthesitis has been lacking. Therefore, this narrative review aims to assess the current knowledge on existing IBD-SpA cohorts, focusing specifically on enthesitis.

## 1. Introduction

Inflammatory bowel diseases (IBDs) are a farraginous agglomerate of chronic and recurrent intestinal illnesses made of two key clinical forms: Crohn’s disease (CD) and ulcerative colitis (UC) [1]. CD features chronic granulomatous transmural inflammation with interrupted lesions grasping any segment of the intestine, ileum, and colon in particular, complicated by intestinal granuloma, obstruction, stricture, and fistula [2], whereas UC is distinguished by incessant mucosal inflammation expanding from the rectum toward the colon without the upper involvement [3].

IBD patients sometimes develop extraintestinal manifestations (EIMs), inflammatory conditions that can involve different organs and systems with a serious impact on morbidity and quality of life [4]. EIMs appear in 25% to 40% of IBD patients and mainly hit the joints, accompanied by the skin, eyes, and hepatobiliary tract [5]. Musculoskeletal symptoms are the most frequent EIM combined with IBD [6]. The Assessment of SpondyloArthritis International Society (ASAS) has proposed classification norms for both types of arthropathy (axial and peripheral), which includes the entire spectrum of spondyloarthritis (SpA), such as IBD-related arthropathy [7]. SpA is a joint title applied to an agglomerate of rheumatic diseases with particular characteristics in common and others distinct from other inflammatory arthritides. It includes IBD-related arthritis, ankylosing spondylitis, reactive arthritis, psoriatic arthritis, and undifferentiated SpA [8].

Decades ago, in an Oxford study [9], it was suggested that peripheral arthritis could be separated into type I, a pauciarticular arthritis, acute and self-limiting (<10 weeks), that commonly accompanies deteriorations of IBD and is not connected with the antigen HLA-B27; and type II, a polyarthritis that manifests as a symmetrical polyarthritis and hits small joints in a progressive manner, with signs commonly lasting for months to years, irrespective of the flow of the intestinal disease. Periarticular manifestations, such as enthesitis, tendinitis, and periostitis, may happen [10,11]. The initial kind of arthritis is associated with disease activity, and healing the underlying IBD is the preferred course of treatment. Conversely, the second type of IBD-associated arthritis often necessitates prolonged therapeutic intervention [12].

Both axial and peripheral manifestations can be present in patients with IBD. In peripheral SpA, arthritis, dactylitis, and enthesitis are the main symptoms. Enthesitis and dactylitis have been investigated less comprehensively in IBD patients than peripheral arthritis. Enthesitis is inflammation at the grip of the tendon, ligament, and joint capsule insertion to bone [13].

Enthesitis, presenting with Achilles tendinitis, plantar fasciitis, and chest wall pain, sometimes leads to structural changes in the underlying bones and causes incapacity [14]. The enthesis has even been presented as a unique organ including functionally linked structures deemed to be the primum movens of the inflammatory process in SpA [15]. The inflammatory involvement of sacroiliac joints and the enthesis appear to be the prime goals in the early phase of SpA [16].

Ultrasound (US) or magnetic resonance imaging (MRI) inspection of the affected area can assist in revealing conditions missed by clinical examination [17]. There are several US and MRI signs of inflammation and destruction of entheses used in diagnosis (Table 1).

The utility of US in the detection of aberrations of fibrocartilaginous entheses in the course of SpA is well known [18]. In 2005, Outcome Measures in Rheumatology (OMERACT) described enthesopathy as the unusual hypoechoic (wastage of usual fibrillar architecture) and/or thickened tendon or ligament at its bony attachment (which can sometimes be presented by hyperechoic foci consistent with calcification), seen in two perpendicular planes that can exhibit a Doppler signal and/or bony changes including enthesophytes or erosions [19].

However, the prevalence of peripheral SpA is frequently underestimated or mistakenly related to corticosteroid overuse, due to transitory manifestation of some oligoarticular forms or the use of chronic corticosteroid treatment [20]. Also, detection of joint and tendon participation could be postponed by the fact that gastroenterologists may not specifically inquire about musculoskeletal signs in daily clinical inspection [21]. Enthesitis is frequently the principal sign of SpA in younger patients [22], but only a small percentage of cases are detected by means of a clinical inspection: for example, in a population-based cohort of 499 Norwegian IBD patients, a clinical examination detected enthesitis in only 11 patients [23]. The pathology of entheses in IBD and SpA patients is frequently underdiagnosed or mistaken for overuse tendon pathology [24]. In a Brazilian study, enthesitis was, interestingly, the only rheumatological symptom in three patients [25], a fact that was also detected by De Vlam et al. [26]. One of these patients was HLA-B27-positive. This is important information as enthesitis is a specific sign of spondyloarthropathies and can be a symptom of one of the diseases of this group, especially in younger patients [27].

Although patients with IBD and SpA appear to have plenty of clinical, immunologic, and genetic characteristics in common [28], the exact connection between these two entities has never been comprehensively defined.

The pathophysiology of enthesitis involves innate and adaptive immunity with an overlap of the interleukin (IL)-17 and IL-23 axis [27]. Accordingly, an abnormal gut microbiome as seen in IBD patients may be a factor in the development of entheseal pathology [29]. Patients with IBD are commonly not examined for enthesitis, and additionally there are no standard proceedings for such examinations. Clinical signs including localized pain, tenderness, and swelling are suggestive of enthesitis [30], yet it is discovered by clinical examination only in a small percentage of patients [31]. The most commonly investigated entheses are those placed in the lower limbs (i.e., plantar fascia, patellar, quadriceps, and Achilles tendons) [17,32].

Enthesitis is the prime lesion in SpA, but an isolated peripheral enthesitis has also previously been detected following vedolizumab therapy in IBD patients [33,34,35]. Previous studies have shown a prevalence of SpA under vedolizumab therapy of around 5% [34,35], but in a cohort of 90 cases, it was actually doubled [36]. This entity, so-called vedolizumab-associated enthesitis, is not an EIM, but it could overlap with entheseal pathology in SpA.

This review describes the current novelties in existing IBD-SpA cohorts, focusing specifically on enthesitis.

## 2. Materials and Methods

In this narrative review, we conducted a search of the PubMed, Scopus, Web of Science, and Cochrane databases. The following terms were used: IBD, CD, UC, and enthesitis.

Manuscripts published from January 2013 to March 2023 were included if they evaluated the prevalence/incidence of enthesitis in cohorts of IBD populations. Articles chosen for inclusion were limited to English-language articles with availabile full texts and clinical studies with adult patients. Meta-analyses, case reports, and reviews were excluded. Article titles were first screened for inclusion by a single investigator (IAO). Abstracts and the full texts were further screened by IAO for definite inclusion and certified by the co-authors (J.V., M.R. and Z.S.). Studies were included if they evaluated the prevalence/incidence of enthesitis in a cohort of patients with IBD.

Data were extracted by a single investigator (IAO) and reviewed by the co-authors. The following data were observed: study setting and design, number of patients with IBD, type of IBD, method of rheumatologic evaluation, and imaging modalities used. The prevalence and incidence of enthesitis in the IBD population, individualized for CD and UC where available, was also collected.

There was no direct patient or public involvement in the study’s design, interpretation, discussion, or the drawing of conclusions.

## 3. Results

**Figure 1:**
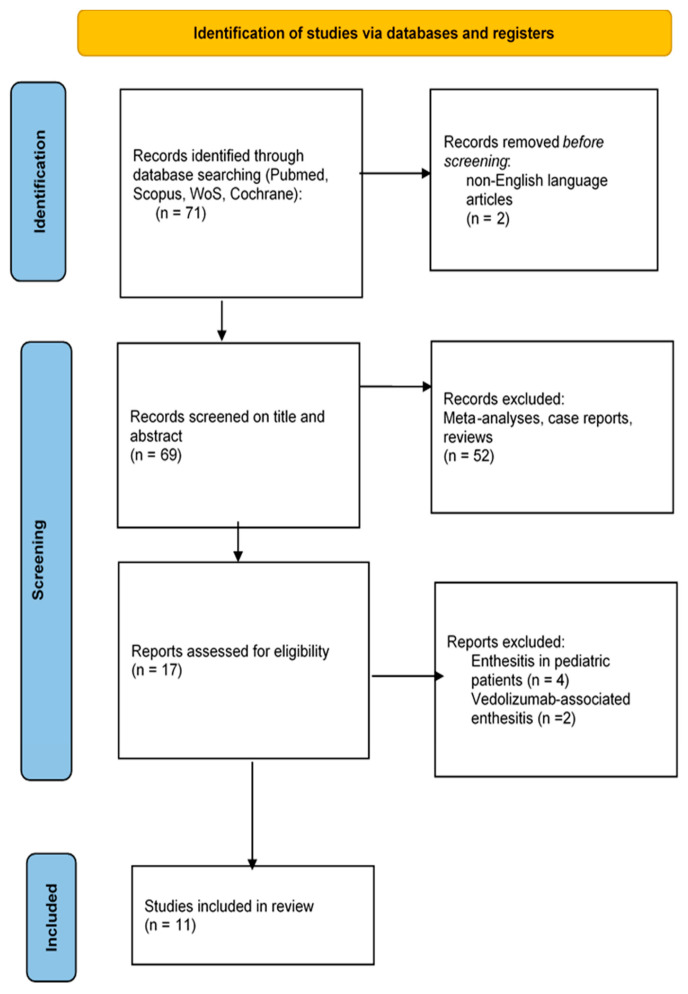
Study selection.

We performed a systematic literature review of studies that evaluated enthesitis in IBD cohorts. The initial database search yielded 71 articles; two were removed because they evaluated vedolizumab-associated enthesitis, and four were removed because they evaluated enthesitis in children. After excluding non-English-language articles, reviews, meta-analyses, and case reports, only 11 manuscripts remained for abstract/full-text assessment, as shown in the PRISMA flow diagram (Figure 1).

Focusing on study design, one study was population-based and ten were clinic-based. Most studies were conducted at a single center, with only two multicenter studies. Three studies reported data for IBD without distinguishing between CD and UC. Rheumatologists were involved in all eleven studies. A physical examination was included in all studies. Other methods of evaluation included questionnaires in 2/11 studies. US was performed in 7/11 studies, and the remaining four studies, in addition to questionnaires, used physical examination, laboratory assessment, and X-ray imaging. Stratified information based on IBD duration was obtained in three studies (Table 2).

Bertolini et al. [37] found clinical evidence of enthesitis (tenderness and/or swelling) in 33% of IBD patients, and it was significantly more frequent in the UC group than the CD group (37.7% versus 25%, *p* = 0.012), which is in discordance with a study conducted by Turkcapar et al. [38]; they found no meaningful differences between UC and CD in regard to the presence of enthesitis.

The study by Bertolini et al. was one of two studies that observed and distinguished between acute and chronic entheseal lesions. Patients with longer disease durations had more frequent entheseal abnormalities at US assessment (85/98 patients (90%) versus 38/53 (72%), *p* = 0.003) [37]. The following entheses were evaluated for tenderness and swelling bilaterally: common extensor tendon (CET), insertion on the lateral epicondyle of the humerus, quadriceps tendon (QT), patellar tendon (PT), tibial tuberosity (TT), Achilles tendon (AT), and plantar fascia (PF) insertion on the calcaneus. US was performed in B-mode and power Doppler (PD) mode. Abnormal findings were entheseal thickening, entheseal hypoechogenicity, bony erosions, enthesophytes, and enlargement of bursae. Entheseal thickening, entheseal hypoechogenicity, and bursal enlargement were considered acute lesions. Bony erosions, calcifications, and enthesophytes were considered chronic lesions. Vascularization was examined using PD mode, and studied at the following zones: cortical bone insertion, body of tendon, and bursa. The detection of vascularization in any of these zones was considered abnormal. Enthesis US vascularity was divided into four distinctive patterns according to the number of vessels involved: 0 = none; 1 = 1–3 vessels; 2 = 4–5 vessels; 3 = >5 vessels. The presence of PD ≥ 1 was considered evincive of an acute lesion [37]. Ultrasonographic discoveries were scored concordant to the Madrid sonography enthesitis index (MASEI) [39] and Glasgow ultrasound enthesitis scoring system (GUESS) [40]. No statistically meaningful differences were detected when comparing patients with shorter or longer disease duration (≤12 months versus >12 months) regarding rheumatological estimation. As expected, groups with longer disease durations (≤12 months versus >12 months) showed more frequent entheseal abnormalities at US assessment, at least one enthesis with erosions, and more entheses with chronic lesions/patient.

In a study by Bakirci Ureyen et al., entheseal inflammation scores were higher in the IBD group than in the healthy control group. They reported data for IBD without distinguishing between CD and UC. Also, IBD duration was independent of inflammation and damage score. Interestingly, they showed BMI correlation to US scores and inflammation in IBD patients [41].

Cantini et al. [42] showed that enthesitis frequency was significantly higher in IBD-SpA patients with associated psoriasis. Enthesitis involvement was not different in patients with UC and those with CD.

Husic et al. [43] did not find a significant correlation of disease duration and activity of IBD with MASEI score, but US-verified enthesitis was more common in patients with IBD than in healthy subjects. No association was found between clinical IBD activity and MASEI, between clinical IBD activity and erosion, nor between PD and enthesophyte subscores.

Bandinelli et al. [44] proposed that enthesitis occurrence can also be present in early IBD. Enthesitis occurrence is connected neither with duration nor with disease activity, concordant with their study. In disagreement with Kiris et al. [45], who showed that entheseal pain tightly correlated with the vascularity shown by PD, 16% of Bandinelli et al.’s patients were positive at PD signal on entheses without symptoms.

Tavassoli et al. [46] found enthesopathy in 6.5% IBD patients without US assessment.

Variola et al. [47] used the IBIS Q (IBd Identification of Spondyloarthritis Questionnaire) for detection of axial SpA and peripheral SpA, but without US examination. The IBIS-Q is a valuable and uncomplicated tool to use in IBD clinics for SpA detection, with a fine statistical performance [47].

In the IBSEN study, during 20 years of disease course, more than every sixth patient had perished from IBD-related peripheral arthritis and every fourth from peripheral spondyloarthritis. Also, a higher proportion of female patients had IBD-related peripheral arthritis and peripheral spondyloarthritis. In 33.1% of patients, peripheral arthritis, dactylitis, and/or enthesitis had happened since the onset of IBD, but enthesitis was diagnosed only when symptoms of inflammation of the insertion of the Achilles tendon to the bone had been present [48].

In the study by Hsiao et al., none of their subjects had peripheral joint pain or swelling, and physical examinations were unremarkable, but regardless of these findings, positive US findings were detected in 13 of the 14 patients [49].

**Table 2:** Study characteristics. Abbreviations: IBD: number of patients with IBD; RI: rheumatologist input; PE: physical examination; US: studies with performed ultrasound examination; PD: power Doppler mode; A/C: studies with US made distinguish between acute vs. chronic lesions; EE: examined entheses; MASEI: Madrid sonography enthesitis index; GUESS: Glasgow ultrasound enthesitis scoring system; ASAS: PD positive entheseal site in ASAS neg. patients; Q: questionnaire; UC: ulcerative colitis; CD: Crohn’s disease; HBI: Harvey–Bradshaw Index; CDAI: Crohn Disease Activity Index; CET: common extensor tendon; QT: quadriceps; PT: patellar tendon; TT: tibial tuberosity; AT: Achilles tendon; PF: plantar fascia; IBIS: IBd Identification of Spondyloarthritis Questionnaire.

Study	Setting	Design	IBD	RI	PE	US	PD	A/C	EE	MASEI	GUESS	ASAS	Q	UC	CD
Variola et al. [47]	clinic	cross-sectional	181	yes	yes	no							IBIS	Partial Mayo score	HBI
Bakici Ureyen et al. [41]	clinic	cross-sectional	43	yes	yes	yes	yes	no	QT, AT, PT, TT	no	no	no	no	no	no
Ossum et al. [48]	population-based	cohort	441	yes	yes	no							yes	no	no
Tavassoli et al. [46]	clinic	cross-sectional	96	yes	yes	no							no	no	no
Bandinelli et al. [44]	clinic	cross-sectional	81	yes	yes	yes	yes	no	QT, AT, PF	no	yes	yes	no	Truelove	CDAI
Bertolini et al. [37]	clinic	cross-sectional	148	yes	yes	yes	yes	yes	CET, QT, PT, TT, AT, PF	yes	yes	yes	no	Mayo	HBI
Husic et al. [43]	clinic	cross-sectional	47	yes	yes	yes	yes	no	QT, PT, AT, PF, TT	yes	no	yes	no	Mayo	CDAI
Cantini et al. [42]	clinic	case–control	88	yes	yes	no							no	no	no
Hsiao et al. [49]	clinic	prospective	18	yes	yes	yes	yes	no	PT, AT, PF	no	yes	yes	no	no	no
Rovisco et al. [50]	clinic	case–control	76	yes	yes	yes	yes	no	PT, AT, PF, QT	no	no	yes	no	Mayo	CDAI
Martinis et al. [51]	clinic	case–control	301	yes	yes	yes	yes	yes	CET, PT, TT, PF, AT	yes	yes	no	no	Mayo	HBI

Rovisco et al. conducted a multicenter study using US assessment to enquire into joint and entheseal involvement in IBD subjects with no signs or symptoms of musculoskeletal disease, and their discoveries signified that the prevalence of sub-clinical entheseal and joint involvement is high in the IBD group [50].

The study by Martinis et al. found that a positive Doppler signal at the entheseal level was more frequently detected in the IBD group with SpA than in IBD patients with fibromyalgia [51].

The predictive value of occult entheseal US abnormalities in IBD subjects for the development of definitive SpA has not yet been investigated in prospective studies. The most affected entheseal site in these studies was the patellar tendon, followed by the Achilles tendon, which is often described as being the most affected site in subjects with SpA [52,53]. The most relevant findings shown by US in the early phase of entheseal pathology were thickness and PD signal at entheses in accordance with previous studies [54,55], probably due to edema, neovascularization, and cell infiltration.

## 4. Discussion

In this narrative review of eleven studies analyzing enthesitis in patients with IBD, several limitations were identified. The majority of the investigated studies were clinic-based, single-center, and cross-sectional in design. This limited number of studies is unlikely to provide sufficient evidence to fully describe the involvement of enthesitis and its correlation with IBD activity. Additionally, there were variations in the scoring systems used to assess IBD activity among these studies, further complicating the interpretation of results.

Also, there were variations in US assessment. While the MASEI included retrocalcaneal and infrapatellar bursitis in the score, bursitis is not taken into account as an elementary lesion of enthesitis according to OMERACT. OMERACT experts were of the opinion that bursae are not a segment of the enthesitis complex, and that inflammation hits them only at a later phase of enthesitis when it expands toward the tendon and peri-tendinous structures [56].

Previous reviews attempted to identify relevant studies about EIMs from database inception to August 2016, and they found only a few estimates available for enthesitis (with a prevalence range from 1% to 54%) [57].

A systematic review by Sakellariou et al. provided an overview of the clinical applicability of musculoskeletal US in subjects with IBD without overt joint involvement, and they included only studies with US assessment. They did not find any correlation between the type of IBD and the disease activity. They discovered that only IBD duration correlated with a higher frequency of US abnormalities, but this result came from a single study that included subjects with very short disease duration (<12 months) [58].

This review represents an attempt to systematically investigate the study design and characteristics of entheseal involvement in IBD patients. Still, it was limited by incomplete or absent reporting of important variables such as body mass index (BMI), medication usage, and heterogeneity in disease activity classification.

Also, a small number of studies performed US assessment, and those that performed it did not all use the same scoring system.

Based on the available data, it is recommended to assess the frequency of enthesitis using both physical examination and ultrasound as the reference standard. However, it is important to acknowledge that data on such assessments are limited. Future studies with larger and more extensive cohorts are necessary to better evaluate enthesitis in IBD patients.

Although peculiar norms for evaluating each disease are accessible, the standard guidelines of seronegative SpA-associated IBD subjects remain to be stated. In particular, some therapeutic options used to control one disease might have a negative implications for other diseases [59].

The relationship between enthesitis and IBD activity index remains unclear, and further research is required to explore this association. It is worth noting that enthesitis may be asymptomatic in the majority of patients, and there is currently a lack of standardized protocols for examining enthesitis in IBD patients. Consequently, many IBD patients are not routinely examined for enthesitis.

The researchers express a particular interest in studies investigating the impact of increased BMI on entheseal involvement in IBD patients, as this information could help prevent further complications. Beneficial treatment is accessible for both axial and peripheral SpA, and precocious diagnosis and treatment are significant to adjust disease progression and decrease the disease burden [60]. Every patient with potential SpA needs to be examined by a rheumatologist for definitive diagnosis, and a combination of multidisciplinary nonpharmacological and pharmacological treatment is essential [61]. For example, in subjects with predominant enthesitis, a biologic treatment can ensure efficacy that supersedes conventional disease-modifying anti-rheumatic medications [62,63,64]. US is rapidly being used in the detection of inflammatory arthritis [65], and it has the power to provide a unique opportunity to assess SpA, particularly in the early disease stage, in IBD.

To the best of our knowledge, this is the first study that systematically highlights the limitations of the available studies on enthesitis in IBD patients, emphasizing the need for more extensive research with standardized protocols. The presence of enthesitis should be assessed using both physical examination and ultrasound, and future studies should aim to clarify the relationship between enthesitis and IBD activity. Additionally, efforts should be made to establish a standard protocol for examining enthesitis in IBD patients, and further investigations into the impact of increased BMI on entheseal involvement are warranted to improve patient outcomes and prevent complications.

Authors should debate the findings and how they can be comprehended from the perspectives of previous studies and of working hypotheses. The results and their implications may be deliberated in the broadest elucidation possible. Future exploration directions should also be highlighted.

## 5. Conclusions

The frequency of the enthesitis should be assessed using both physical examination and ultrasound as the reference standard. Data on such assessments are limited. Future trials with more comprehensive cohorts are required to assess enthesitis. Is enthesitis generally indicative of IBD activity? Enthesitis can be asymptomatic in the majority of patients. IBD patients are often not examined for enthesitis, and there is a lack of a standard protocol. Many gastroenterologists lack the experience to detect or distinguish inflammatory from degenerative musculoskeletal disease or fibromyalgia. Conversely, rheumatologists are usually not confident in their competence to differentiate IBD from other gastrointestinal manifestations such as irritable bowel syndrome or coeliac disease.

It is notable that rheumatologists and gastroenterologists have become aware of both facets of the disease in order to actively search for signs that will induce further investigation and prompt diagnosis. Given the lack of scientific proof, partnership between physicians of both specialties is obligatory so that patients receive suitable treatments for their illnesses and are followed up properly.

We look forward to seeing some studies about the impact of increased BMI on entheseal involvement in IBD patients in order to prevent further complications. The use of cheaper and faster imaging techniques, such as US, could be routinely represented in everyday clinical examination to estimate occult SpA accurately, thus preventing incapacity and deterioration of quality of life in IBD subjects.

Collaboration between rheumatologists and gastroenterologists will provide an integral evaluation of IBD subjects. The main purpose of this collaboration should be prompt diagnosis and tailored treatment of EIMs in order to ameliorate the quality of life of these subjects.

## Figures and Tables

**Table 1:** US and MRI signs of enthesitis.

US	MRI
fluid in paratenon	fluid in paratenon
vascularisation	bone edema
peri-entheseal soft-tissue edema	entheseal edema
altered echogenicity	entheseal enhancement
thickening of tendon	peri-entheseal soft-tissue edema
erosion	erosion
cortical roughening	cortical roughening
enthesophyte	enthesophyte

## Data Availability

Not applicable.
